# Transcatheter Repair and Replacement Technologies for Mitral Regurgitation: a European Perspective

**DOI:** 10.1007/s11886-021-01556-6

**Published:** 2021-07-16

**Authors:** Joris F. Ooms, Nicolas M. Van Mieghem

**Affiliations:** Department of Interventional Cardiology, Thoraxcenter, Erasmus University Medical Center, Office Nt 645, Dr. Molewaterplein 40, 3015 GD Rotterdam, The Netherlands

**Keywords:** Mitral valve regurgitation, Percutaneous repair, Edge-to-edge coaptation, Annuloplasty, Transcatheter replacement, Advanced pre-procedural planning

## Abstract

**Purpose of Review:**

We aimed to picture the contemporary landscape of available catheter-based repair and replacement solutions for mitral regurgitation (MR) in Europe.

**Recent Findings:**

Edge-to-edge repair remains the dominant technique for transcatheter mitral valve repair especially in the context of secondary mitral regurgitation. Two recent randomized trials reported seemingly contradicting clinical results with transcatheter edge-to-edge repair for patients with heart failure and severe secondary MR. A proportionality framework related to secondary MR was proposed to help explain inconsistencies but requires further research. (In)Direct annuloplasty primarily aims to correct secondary MR; however, the scientific basis seems less robust. One dedicated transcatheter heart valve has the CE mark for mitral valve replacement but requires transapical access. Balloon-expandable transcatheter aortic valve platforms are emerging for transvenous transseptal mitral replacement in the context of mitral annular calcification, a failing surgical mitral bioprosthesis, or annuloplasty. Advanced computed tomography imaging techniques improved pre-procedural planning and introduced the option for modeling and simulation.

**Summary:**

Development of a toolbox of catheter-based technologies, complementary imaging modalities, and refined patient selection offer novel perspectives to high-risk patients with primary or secondary MR. Clinical trials are required to help formulate evidence-based guidelines for the management of mitral valve disease.

## Introduction

In Europe, native mitral valve disease is the second most referred etiology for surgical or transcatheter intervention, only surpassed by aortic valve disease. A recent European survey revealed that mitral valve regurgitation and stenosis comprised 21% and 5% of all referrals for valve interventions [1]. The latest guidelines of the European Society of Cardiology (ESC) dating from 2017 predominantly recommend surgical management of severe—in particular—primary or degenerative mitral regurgitation (PMR) [2]. For secondary or functional mitral valve regurgitation (SMR), randomized controlled trials failed to prove the clinical benefit of surgical mitral repair (or replacement) and currently there are no strong guideline recommendations for surgical treatment of isolated SMR [2–5]. Conversely, continued device iterations revolutionized catheter-based treatment alternatives to mitral surgery. In particular, landmark trials underscored the value of transcatheter edge-to-edge repair for functional mitral regurgitation in the context of heart failure with reduced ejection fraction (HFrEF) [6••, 7••]. Importantly, novel 3D imaging tools including computed tomography-derived simulation and printing emerged, offering unprecedented insights and complementing pre-procedural planning of transcatheter mitral valve procedures.

Herein, we review the contemporary European landscape of transcatheter mitral valve repair and replacement (TMVr and TMVR) technologies, mainly in the setting of mitral regurgitation. Additionally, we briefly discuss future perspectives and highlight the importance of advanced imaging tools.

### Mitral Valve Repair

A plethora of catheter-based technologies (Table 1) have been developed which may mimic the toolbox of techniques and skills at a cardiac surgeon’s disposal. The distinction between primary (or degenerative) and secondary (or functional) mitral regurgitation is relevant and may determine which technique would be most suitable for a particular indication. Clearly, the importance and maturation of certain concepts may be different in the context of (conventional) surgery vs. catheter-based solutions. Hence, fundamentally different principles apply in surgery vs. the interventional reality. Indeed, annuloplasty is the dominant concept for repair in surgery as opposed to transcatheter edge-to-edge repair technique outside of the operating room.

**Table 1:**
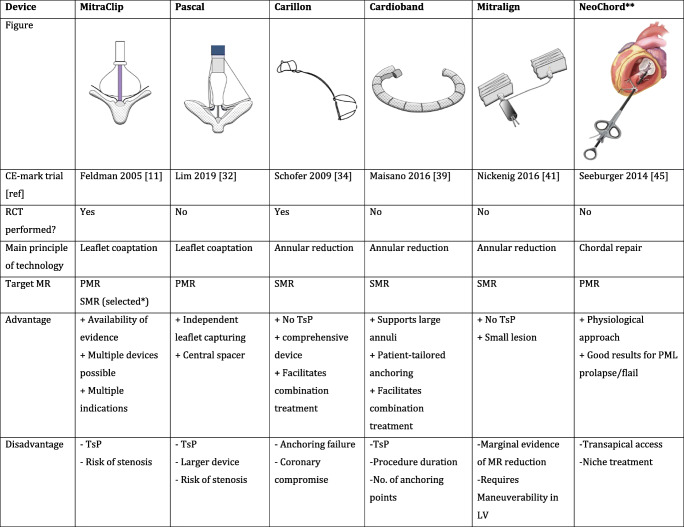
Currently available devices for transcatheter mitral valve repair with a CE mark

*Based on COAPT criteria: patients on maximized HF therapy including CRT and with large SMR in relation to LV dimensions

**NeoChord figure is used with permission from NeoChord, Inc.

*RCT* randomized controlled trial, *PMR* primary mitral regurgitation, *SMR* secondary mitral regurgitation, *TsP* transseptal puncture, *LV* left ventricle

In general, specific technologies may have different and device-specific anatomical targets. Apart from selection and reporting bias, nonuniform definitions were applied in the different safety and feasibility trials and hamper formal comparison of transcatheter techniques. A consensus statement on study endpoints and definitions may help clarify fundamental outcome differences between mitral valve technologies moving forward [8]. Additionally, a time-bias should be ascertained. Trailblazing technologies may face unique challenges and lead to essential learning that may also serve forthcoming devices.

## Edge-to-Edge Leaflet Coaptation

### MitraClip

Ottavio Alfieri pioneered the surgical edge-to-edge leaflet coaptation technique that lends itself to a transformation into completely percutaneous, transvenous transseptal catheter-based designs [9, 10]. Arguably, MitraClip (Abbott Vascular) was the first of its kind and introduced a clip with two arms and inner U-shaped grippers to capture and lock mitral leaflets. MitraClip clinical safety and feasibility was demonstrated in the EVEREST I trial [11]. CE mark was obtained in 2008. The randomized EVEREST II trial compared MitraClip with surgical repair for (predominantly) primary MR and demonstrated superior 30-day safety with MitraClip driven by less bleedings and similar short-term mortality rates (1% vs. 2% in MitraClip vs. surgery). However, MR reduction appeared less effective with MitraClip. At 1 year, more than mild MR (≥2+) was present in 45% of patients with MitraClip vs. 17% in the surgical arm and 20% of MitraClip patients needed mitral surgery vs. 2% of the surgical cohort [12]. Long-term follow-up confirmed higher rates of grade 3+/4+ MR and more need for mitral surgery after MitraClip [13, 14].

Postapproval registries focused more on secondary MR. The ACCESS-EU registry reported clinical safety and efficacy of MitraClip in a high-risk population predominantly suffering from SMR (69%) [15]. MR grade ≤2+ was achieved in 91% of patients immediately post clipping and in 79% at 12 months. Additionally, functional status significantly improved at 1-year follow-up. Other registries on patients with SMR at high operative risk identified procedural success, LV dysfunction, LV dilatation, and advanced heart failure (HF) as predictors for mortality at 1 year [16–18]. Based on these registry data, the latest European guidelines on valvular disease considered catheter-based edge-to-edge MVr in patients with SMR with a favorable anatomy, at high operative risk, and on guideline-directed heart failure therapy a class IIb recommendation [2]. The 2020 ACC/AHA guidelines for the management of valvular heart disease formulated more firm class IIa recommendations for transcatheter edge-to-edge repair in the context of primary severe MR and high or prohibitive surgical risk or chronic severe secondary MR with depressed LV function (EF ‹ 50%) despite optimal guideline-directed medical therapy [19••].

Two randomized controlled trials evaluated the effect of MitraClip in patients with HFrEF and moderate to severe SMR on top of (optimal) heart failure treatment and reported seemingly contradicting results in terms of clinical outcomes [6••, 7••]. The Cardiovascular Outcomes Assessment of the MitraClip Percutaneous Therapy for Heart Failure Patients with Functional Mitral Regurgitation (COAPT) trial showed a convincing treatment benefit of MitraClip but the Percutaneous Repair with the MitraClip Device for Severe Functional Mitral Regurgitation (MITRA-FR) trial showed no difference. The trial designs provided explanations for this discrepancy. HF therapy was scrutinized prior to study enrolment in COAPT, whereas HF medical optimization continued throughout the trial in MITRA FR. MR cutoff to enter the study was based on American guidelines in COAPT (i.e., EROA › 0.4 cm^2^) [20] and on European guidelines in MITRA FR (i.e., EROA › 0.2 cm^2^) [21]. COAPT also introduced an upper limit of LV dilatation. These nuances resulted in fundamentally different patient phenotypes and study populations characterized by more LV dilatation and less severe MR in MITRA FR as compared to less dilated but more severe MR in COAPT [6••, 7••, 22]. The “(dis)proportionality theory” was proposed to help reconcile the different clinical outcomes: in disproportionate SMR, the severity appears more extensive than what would be expected based on LV dilatation and EF and would reflect asymmetrical LV dysfunction (due to segmental or global dyssynchrony) that is also imposed on the mitral apparatus [23•]. A high ratio of EROA and LV end-diastolic volume (LVEDV) was proposed as a marker for disproportionality. SMR was deemed disproportionate in COAPT but more proportionate in MITRA FR and MitraClip might be particularly effective to reduce disproportionate SMR [24]. However, the EuroSMR registry failed to confirm the value of this proportionality framework in a cohort of 1016 patients with depressed LV function and moderate to severe SMR [25].

Arguably, patients in every day practice may not resemble the highly selected patient population of the COAPT trial in which only 39% of screened patients were enrolled in the trial. Also, continued implementation of guideline-directed medical therapy is challenging outside of a clinical trial as was pointed out by incomplete adherence to medical therapy in US and Dutch registries [26, 27]. Lastly, echocardiography studies in routine clinical practice may not allow the application of the disproportionality concept because key quantitative measurements often are not recorded in the clinic.

Regardless, the 2020 ACC/AHA Guidelines on the management of valvular disease reinforced the COAPT echocardiography criteria and defined appropriate anatomy as EF 20–50%, LV end-systolic diameter ≤ 70 mm and pulmonary artery pressure ≤ 70 mmHg [19••]. Additionally, the ESC recently released a consensus document advocating MitraClip in patients who would fit within the COAPT inclusion criteria [28]. Another report highlighted the value of MitraClip in patients with moderate to severe SMR with end-stage heart failure awaiting heart transplantation or LVAD [29]. Recent device iterations (i.e., MitraClip G4) feature separate grasping ability and different arm/gripper width, and facilitate real-time LA-pressure monitoring on the guide catheter. Initial results of a study including 59 patients showed residual MR (grade › 2+) in 3.5%, which appears to be surpassing the results of large trials in terms of MR grade reduction [30]. In particular for SMR, wider arms might contribute to enhanced leaflet apposition as none of the patients in the SMR arm had SMR grade › 2 at 30 days follow-up.

### Pascal

The Pascal system (Edwards, Irvine, CA) achieves edge-to-edge leaflet repair around a central spacer using paddles and clasps [31]. The CLASP study confirmed device safety and efficacy in a study comprising 62 patients of which 36% and 56% had PMR and SMR respectively [32]. Procedural success rate was 92% and MR was reduced to grade ≤2+ at 30 days in 98% of patients and was maintained out to 1 year [33]. PASCAL Ace is the latest iteration that introduces more size options with a smaller central spacer.

PASCAL CLASP IID/IIF trial (NCT03706833) is an ongoing randomized trial aiming to enroll 1275 patients with PMR at prohibitive operative risk or SMR on guideline-directed medical therapy to edge-to-edge repair with PASCAL or MitraClip. The primary endpoint of this noninferiority trial is [1] composite of major adverse events at 30 days; [2] MR severity reduction at 6 months; (3) recurrent heart failure hospitalization and all-cause mortality at 2 years.

## (In)Direct Annuloplasty

Annuloplasty is the cornerstone of mitral repair in the surgical toolbox. Various catheter-based concepts have been developed to create direct or indirect annuloplasty primarily aiming to correct SMR. Several catheter-based systems have been developed and will be reviewed in the following sections.

### Carillon

The Carillon Mitral Contour System (Cardiac Dimensions, Inc., Kirkland, WA) consists of a nitinol ribbon with a proximal (larger) and distal (smaller) anchor to be seated close to the orifice of the coronary sinus and in the great cardiac vein respectively. There are 37 different device sizes available based on the combination of proximal and distal anchor and ribbon dimensions. Sizing is primarily based on coronary sinus angiography. The device uses a transjugular venous approach and is deployed in the coronary sinus and subsequently bended into a C-shape, cinching the peri-annular tissue to indirectly reduce mitral annular dimensions (i.e., indirect annuloplasty). Device safety and feasibility was studied in the AMADEUS (*n* = 48) [34] and TITAN trials (*n* = 53) [35–37]. The REDUCE-FMR trial was a randomized sham-controlled study comparing the Carillon device (*n*=87) with sham controls (*n* = 33) [38]. Successful device deployment was achieved in 73/87 patients. The primary endpoint of change in mitral regurgitant volume at 12 months was significantly better in the treatment arm compared to the sham group. LV volumes were also significantly reduced. Of note, the study was limited by a substantial number of patients with SMR grade ‹ 2+ at baseline (30%) and a low number of patients in the control group with available transthoracic echocardiogram (TTE) at 1 year follow-up (39%). One-year mortality was 13% vs. 15% in treatment vs. control group, but the study was not powered to assess differences in clinical endpoints or QoL.

### Cardioband

The Cardioband system (Edwards Lifesciences, Irvine, CA) consists of a polyester sleeve that is connected to 12 to 17 helical stainless steel anchors. The anchors are inserted through a transseptal approach at the atrial side of the mitral annulus starting at the anterior commissure and moving in 8-mm increments along the posterior annulus toward the posterior commissure. A specific size adjustment tool allows approximating the anchors and cinch and remodelling the annulus and creating a form of direct annuloplasty. Early feasibility and safety studies reported acceptable safety and efficacy out to 1 year in 60 patients [39, 40]. Mitral Valve Research Consortium (MVARC) defined technical, device, and procedural success was achieved in 97%, 72%, and 68% respectively (the latter two assessed at 30 days). MR recurrence after successful initial reduction was observed in 22% of patients at 1 year. Salient device-related complications were pericardial effusion or tamponade, left circumflex artery damage, and partial anchor disengagement. At discharge, MR grade ≤moderate was present in 88% of patients. Mitral application is currently limited and the concept is now predominantly being explored for tricuspid repair.

### Mitralign

The Mitralign system (Mitralign, Tewksbury, MA) includes two sets of pledgets which are attached to the posterior mitral annulus (segments P1/P3). A connecting suture is subsequently used to plicate the annulus. The insertion requires a retrograde transaortic approach [41]. The safety and feasibility study reported successful implantation in 71% of patients and SMR improvement in 50% of patients at 6 months. Small changes in mitral annular diameters, coaptation length, and tenting distance were reported, but quantitative parameters of regurgitation did not change significantly. Currently, the Mitralign concept has shifted toward potential application in tricuspid repair [42].

## Chordal Repair

Ruptured chordae with subsequent flail leaflet often contribute to significant PMR. Surgical repair would include chordal replacement, annuloplasty, and other surgical techniques [2, 43, 44]. Multiple catheter-based chordal repair solutions are under development and one design has the CE mark.

### NeoChord

The NeoChord DS1000 device (NeoChord, Inc., Minneapolis, MN) encompasses a transapically inserted shaft with a grasping mechanism to capture the prolapsing/flail segment. An incorporated needle then punctures the leaflet and a NeoChord is retracted and fixated in the LV apex [45]. In the proof of concept TACT trial, 26/30 patients with severe PMR due to isolated prolapse of the posterior leaflet (PML) received ≥1 NeoChord [45]. Residual MR ≤2+ at 30 days was achieved in 59% of the entire cohort and in 63% and 86% for the subgroups with multiple chordae and postolateral access respectively. An European registry including 213 low-risk patients found an overall procedural success in 97% of patients and confirmed that particularly PML subtypes of flail/prolapse were responsive to this approach [46].

## Future Developments in Mitral Valve Repair

The analogy with surgical repair will continue, and expectedly various catheter-based mitral concepts could be combined to improve overall repair result, minimize residual MR, and maintain the effect over time. Reports already illustrated the complementary effects of edge-to-edge repair with (in)direct annuloplasty or chordal repair [47–50]. The optimal sequence of combining techniques remains unsettled, although intuitively it would make sense to first proceed with annuloplasty and improve leaflet coaptation in order to facilitate leaflet approximation with edge-to-edge repair afterwards. The optimal treatment sequence will need further study in randomized clinical trials.

Numerous types of catheter-based devices for MVr are under development. Some rely on familiar mechanisms to treat MR [51, 52], while others introduce novel approaches [53]. Clearly, a toolbox of catheter-based techniques is shaping up to eventually determine a patient-tailored selection on a case by case basis.

## Mitral Valve Replacement

Current European guidelines recommend surgical mitral valve replacement (MVR) if durable MVr is not feasible [2]. Mitral valve replacement typically comes with a higher operative risk [54]. Transcatheter MVR (TMVR) faces specific challenges related to device anchoring and LV outflow tract obstruction (LVOTO) [8]. Advanced pre-procedural planning including simulation may refine patient selection, enhance procedure safety, and improve overall TMVR outcome.

### Tendyne

The self-expandable Tendyne Mitral Valve System (Abbott Vascular, Chicago, IL) is the sole CE-marked dedicated TMVR technology and consists of a D-shaped outer sealing stent and a circular inner stent that houses a trileaflet valve. The prosthesis requires transapical access and is connected to the LV apex using a tether and an epicardial pad [55]. A first safety and feasibility study demonstrated favorable results in a selected cohort of 30 patients with SMR at high operative risk [55]. Successful deployment was achieved in 93%, mild paravalvular leak (PVL) was noted in 1 patient, and freedom from major adverse events was 83%. Indexed LV end-diastolic volumes (LVEDV) decreased significantly and QoL scores improved. An extended high-risk cohort of 100 patients (89% SMR) documented a 96% technical success rate [56]. Thirty-day and 1-year mortality were 6% and 26% respectively. Interestingly, leaflet thrombosis was observed in 6 patients and justified a protocol change with vitamin K antagonist antithrombotic therapy post TMVR. TTE follow-up showed that MR was absent in 98% and both LVEDV and LVEF decreased. Tendyne offers a valuable solution for MR patients with large annuli unable to undergo surgery or transcatheter MVr (Table 2).

**Table 2:**
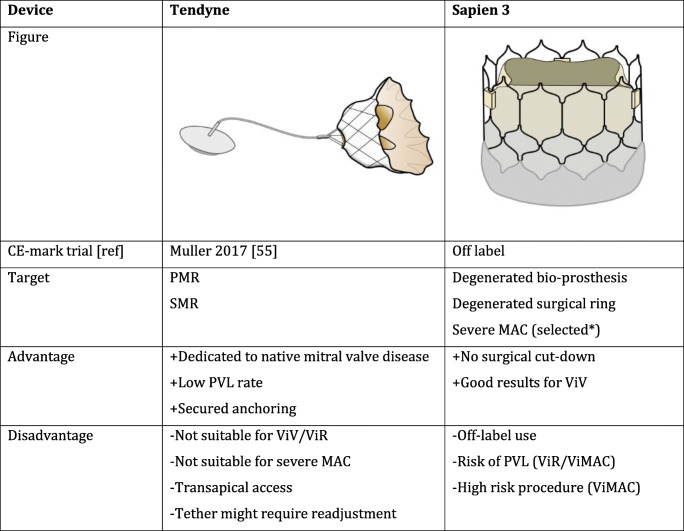
Commercially available devices for transcatheter mitral valve replacement

*Circumferential calcium, low risk of LVOT obstruction

*PMR* primary mitral regurgitation, *SMR* secondary mitral regurgitation, *ViV* valve-in-valve, *ViR* valve-in-ring, *ViMAC* valve-in-mitral annular calcification

Feasibility of the Tendyne system in excessively calcified mitral annuli (MAC) was examined in a small compassionate use study of 9 patients [57]. MVARC-defined technical success was achieved in 8/9 patients, with one patient requiring alcohol septal ablation due to LVOTO. All patients were discharged with no residual MR. Although candidates were carefully selected based on CT-assessed anatomical parameters, these results hold promise for the treatment of a challenging MR/MS phenotype. A substudy of the prospective SUMMIT trial might be able to verify these results in a larger cohort (NCT03433274).

### Balloon-Expandable TMVR

The balloon-expandable Sapien 3 transcatheter heart valve (Edwards Lifesciences, Irvine, CA, USA) is commercially available for aortic valve replacement, but is used off-label for TMVR in the context of a failing mitral bioprosthesis (ViV), valve in surgical ring (ViR), or valve in mitral annulus calcification (MAC) (ViMAC) [58–60]. Two multicenter registries reported on outcomes of TMVR with the balloon-expandable SAPIEN platform in patients with severe MR, MS, or a combination at high or prohibitive operative risk [58, 59]. MS was the primary indication for TMVR in the ViMAC group (Table 3). Interestingly, the ViV cohort outperformed the ViR and ViMAC cohorts in terms of technical, device, and procedural success, mainly attributed to more consistent and robust anchoring possibilities limiting the risk of device migration or embolization. Additionally, the fixed frame of the surgical bioprosthesis, without a dominant (native) AML, may mitigate the LVOTO risk. In contrast, ViMAC had the lowest procedural success rate of 41–49% and 30-day mortality varied between 18 and 22%. MAC may feature a more complex, asymmetrical annulus with noncircular calcification that may complicate the implant of a circular transcatheter heart valve and explain the increased risk for embolization or residual PVL. Furthermore, the risk for LVOTO is higher with ViMAC as the irregular shape of the calcified native anatomy could force the Sapien valve to shift toward the LVOT during deployment. Also, protrusion of the Sapien into the LV could result in a deflection of the AML toward a narrowed LVOT causing obstruction. A kissing balloon technique with simultaneous inflation of the SAPIEN THV and a balloon in the LVOT may facilitate THV positioning and also secure the neo-LVOT by slightly pivoting the THV away from the outflow tract [61]. ViR technical success varied between 57 and 60% with a 5% LVOTO rate and residual ≥ moderate MR in 9–13% [58]. Ring type (complete vs. incomplete and rigidity) may determine procedure success [59]. One-year mortality of the ViR and ViMAC groups was high and attests to morbidity of these groups. Advanced pre-procedural workup may refine patient selection and dedicated devices might further increase procedure safety and survival.

**Table 3:** Data of two major TMVR registries

Registry	Yoon et al. 2019a			Guerrero et al. 2020b		
Number of patients	521			903		
-ViV	322			680		
-ViR	141			123		
-ViMAC	58			100		
Age	73 ± 12			75 [67-82]		
STS-PROM (%)	9 ± 7			10 [7-16]		
Sapien valve (%)	90			96		
Access						
-Transseptal (%)	40			43		
-Transapical (%)	60			45		
Outcomes	ViV (%)	ViR (%)	ViMAC (%)	ViV (%)	ViR (%)	ViMAC (%)
Technical success*	94	81	62	91	83	74
LVOTO**	2	5	40	1	5	10
Conversion to surgery	1	3	9	1	2	2
MR grade ≥2+ (30d)	3	13	13	2	9	6
Device success (30d)*	85	70	53	84	68	59
Procedural success (30d)*	74	57	41	76	60	49
Mortality						
-30 days	6	10	18	8	12	22
-1 year	14	31	63	-	-	-

^a.^Yoon SH et al. Transcatheter Mitral Valve Replacement for Degenerated Bioprosthetic Valves and Failed Annuloplasty Rings. Eur Heart J. 2019

^b.^Guerrero M et al. Thirty-Day Outcomes of Transcatheter Mitral Valve Replacement for Degenerated Mitral Bioprostheses (Valve-in-Valve), Failed Surgical Rings (Valve-in-Ring), and Native Valve With Severe Mitral Annular Calcification (Valve-in-Mitral Annular Calcification) in the United States. Circ Cardiovasc Interv. 2020

*MVARC defined** Different definitions were used: Yoon at al. Obstruction: › 10 mmHg gradient, Guerrero et al. Obstruction: Hemodynamic compromise

*ViV* valve-in-valve, *ViR* valve-in-ring, *ViMAC* valve-in-mitral annular calcification, *STS-PROM* Society of Thoracic Surgeons Predicted Risk Of Mortality, *LVOTO* left ventricular outflow tract obstruction, *30d* 30 days

## Future Developments in TMVR

### Development of Dedicated Transcatheter Mitral Valves

Numerous TMVR concepts are under development introducing unique anchor mechanisms leveraging the mitral leaflets, sub-annular apparatus, or a docking platform [62]. Several prospective studies are ongoing and could help redefine the palette of catheter-based therapies for MR and the role of TMVR in particular (NCT03039855, NCT03242642) [62].

### Advanced Pre-procedural Planning

Mitral valve surgery typically has relied on imaging workup based on conventional radiography and echocardiography. TMVR-specific procedural risks such as valve embolization or LVOTO have detrimental consequences. Computed tomography has become an essential complementary imaging tool for TMVR. Meticulous planning of access, device type/size, anchoring, and the effects on the LVOT are crucial [63]. Advanced CT-derived imaging allows creating patient-specific 3D computational models (3DCMs). These models visualize spatial relations of the heart and allow detailed 3D measurements of the mitral annulus and apposing structures. Furthermore, virtual TMVR allows appreciation of the device landing zone and the risk for embolization/PVL/LVOTO [64]. An important concept is the neo-LVOT, which is the geometrically modified left ventricular outflow tract created after implantation of a transcatheter heart valve in mitral position caused by valve protrusion in the LV and deflection of the AML toward the LVOT [65]. Virtual 3DCMs that incorporate bioprosthetic valve type, size, and implantation depth could potentially reduce TMVR-associated LVOTO risk. Subsequent 3D-printing or assessment of 3DCM in virtual reality could lead to improved understanding of the targeted disease.

Assessment of the (neo-)LVOT with CT-derived 3DCMs was originally performed in models derived from a single (i.e., end-systolic) phase in the cardiac cycle [63]. However, with increased experience in TMVR, it became apparent that pre-procedural planning based on a single phase might be misleading and generate inaccurate information on anatomical dimensions and device/host relationships. Indeed, geometrical LVOTO as determined in end-systole may be clinically irrelevant because most of the stroke volume is ejected earlier in systole and LVOT could therefore be larger [66]. Full-cycle evaluation of the neo-LVOT in 3DCM (i.e., 4D models) using automated segmentation software may optimize pre-procedural planning and further improve patient selection for TMVR.

Existing CT software packages do not implement device/host interactions. New technology allows simulation of conformational changes and deformations post TMVR by accounting for tissue (host) and device characteristics/properties [67]. These simulations could provide detailed information on post-deployment valve integrity, native calcium displacement, and neo-LVOT shape. In the future, simulations might also provide algorithms to compute pressure gradients and flow patterns and predict more accurately PVL and/or hemodynamic compromise [68, 69].

## Conclusion

The development of a toolbox of catheter-based technologies, new complementary imaging modalities, and refined patient selection offer new perspectives to (elderly) patients with primary or secondary mitral valve disease. More research and clinical trials are required to help formulate evidence-based guidelines for the role of catheter-based management of mitral valve disease.

## References

[1] Iung B, Delgado V, Rosenhek R (2019). Contemporary Presentation and Management of Valvular Heart Disease: The EURObservational Research Programme Valvular Heart Disease II Survey. Circulation.

[2] Baumgartner H, Falk V, Bax JJ (2017). 2017 ESC/EACTS Guidelines for the management of valvular heart disease. Eur Heart J.

[3] Acker MA, Parides MK, Perrault LP (2014). Mitral-valve repair versus replacement for severe ischemic mitral regurgitation. N Engl J Med.

[4] Goldstein D, Moskowitz AJ, Gelijns AC (2016). Two-year outcomes of surgical treatment of severe ischemic mitral regurgitation. N Engl J Med.

[5] Michler RE, Smith PK, Parides MK (2016). Two-year outcomes of surgical treatment of moderate ischemic mitral regurgitation. N Engl J Med.

[6.••] Stone GW, Lindenfeld J, Abraham WT (2018). Transcatheter mitral-valve repair in patients with heart failure. N Engl J Med.

[7.••] Obadia JF, Messika-Zeitoun D, Leurent G (2018). Percutaneous repair or medical treatment for secondary mitral regurgitation. N Engl J Med.

[8] Stone GW, Adams DH, Abraham WT (2015). Clinical trial design principles and endpoint definitions for transcatheter mitral valve repair and replacement: part 2: endpoint definitions: a consensus document from the Mitral Valve Academic Research Consortium. Eur Heart J.

[9] Maisano F, Torracca L, Oppizzi M (1998). The edge-to-edge technique: a simplified method to correct mitral insufficiency. Eur J Cardiothorac Surg.

[10] Alfieri O, Maisano F, De Bonis M (2001). The double-orifice technique in mitral valve repair: a simple solution for complex problems. J Thorac Cardiovasc Surg.

[11] Feldman T, Wasserman HS, Herrmann HC (2005). Percutaneous mitral valve repair using the edge-to-edge technique: six-month results of the EVEREST Phase I Clinical Trial. J Am Coll Cardiol.

[12] Feldman T, Foster E, Glower DD (2011). Percutaneous repair or surgery for mitral regurgitation. N Engl J Med.

[13] Feldman T, Kar S, Elmariah S (2015). Randomized comparison of percutaneous repair and surgery for mitral regurgitation: 5-year results of EVEREST II. J Am Coll Cardiol.

[14] Takagi H, Ando T, Umemoto T, Group A (2017). A review of comparative studies of MitraClip versus surgical repair for mitral regurgitation. Int J Cardiol.

[15] Maisano F, Franzen O, Baldus S (2013). Percutaneous mitral valve interventions in the real world: early and 1-year results from the ACCESS-EU, a prospective, multicenter, nonrandomized post-approval study of the MitraClip therapy in Europe. J Am Coll Cardiol.

[16] Capodanno D, Adamo M, Barbanti M (2015). Predictors of clinical outcomes after edge-to-edge percutaneous mitral valve repair. Am Heart J.

[17] Puls M, Lubos E, Boekstegers P (2016). One-year outcomes and predictors of mortality after MitraClip therapy in contemporary clinical practice: results from the German transcatheter mitral valve interventions registry. Eur Heart J.

[18] Glower DD, Kar S, Trento A (2014). Percutaneous mitral valve repair for mitral regurgitation in high-risk patients: results of the EVEREST II study. J Am Coll Cardiol.

[19.••] Otto CM, Nishimura RA, Bonow RO (2021). 2020 ACC/AHA Guideline for the management of patients with valvular heart disease: executive summary: A Report of the American College of Cardiology/American Heart Association Joint Committee on Clinical Practice Guidelines. J Am Coll Cardiol.

[20] Zoghbi WA, Adams D, Bonow RO (2017). Recommendations for Noninvasive evaluation of native valvular regurgitation: a report from the American Society of Echocardiography Developed in Collaboration with the Society for Cardiovascular Magnetic Resonance. J Am Soc Echocardiogr.

[21] Lancellotti P, Tribouilloy C, Hagendorff A (2013). Recommendations for the echocardiographic assessment of native valvular regurgitation: an executive summary from the European Association of Cardiovascular Imaging. Eur Heart J Cardiovasc Imaging.

[22] Asch FM, Grayburn PA, Siegel RJ (2019). Echocardiographic outcomes after transcatheter leaflet approximation in patients with secondary mitral regurgitation: the COAPT Trial. J Am Coll Cardiol.

[23.•] Grayburn PA, Sannino A, Packer M (2019). Proportionate and disproportionate functional mitral regurgitation: a new conceptual framework that reconciles the results of the MITRA-FR and COAPT Trials. JACC Cardiovasc Imaging.

[24] Packer M, Grayburn PA (2019). Contrasting effects of pharmacological, procedural, and surgical interventions on proportionate and disproportionate functional mitral regurgitation in chronic heart failure. Circulation.

[25] Orban M, Karam N, Lubos E, Kalbacher D, Braun D, Deseive S, et al. Impact of Proportionality of Secondary Mitral Regurgitation on Outcome After Transcatheter Mitral Valve Repair. JACC Cardiovasc Imaging. 2021;14(4):715–25. 10.1016/j.jcmg.2020.05.042 32861652

[26] Greene SJ, Butler J, Albert NM (2018). Medical therapy for heart failure with reduced ejection fraction: The CHAMP-HF Registry. J Am Coll Cardiol.

[27] Brunner-La Rocca HP, Linssen GC, Smeele FJ (2019). Contemporary drug treatment of chronic heart failure with reduced ejection fraction: the CHECK-HF Registry. JACC Heart Fail.

[28] Seferovic PM, Ponikowski P, Anker SD (2019). Clinical practice update on heart failure 2019: pharmacotherapy, procedures, devices and patient management. An expert consensus meeting report of the Heart Failure Association of the European Society of Cardiology. Eur J Heart Fail.

[29] Godino C, Munafo A, Scotti A (2020). MitraClip in secondary mitral regurgitation as a bridge to heart transplantation: 1-year outcomes from the International MitraBridge Registry. J Heart Lung Transplant.

[30] Chakravarty T, Makar M, Patel D (2020). Transcatheter edge-to-edge mitral valve repair with the MitraClip G4 System. JACC Cardiovasc Interv.

[31] Praz F, Spargias K, Chrissoheris M (2017). Compassionate use of the PASCAL transcatheter mitral valve repair system for patients with severe mitral regurgitation: a multicentre, prospective, observational, first-in-man study. Lancet.

[32] Lim DS, Kar S, Spargias K (2019). Transcatheter valve repair for patients with mitral regurgitation: 30-day results of the CLASP Study. JACC Cardiovasc Interv.

[33] Webb JG, Hensey M, Szerlip M (2020). 1-Year outcomes for transcatheter repair in patients with mitral regurgitation from the CLASP Study. JACC Cardiovasc Interv.

[34] Schofer J, Siminiak T, Haude M (2009). Percutaneous mitral annuloplasty for functional mitral regurgitation: results of the CARILLON Mitral Annuloplasty Device European Union Study. Circulation.

[35] Siminiak T, Wu JC, Haude M (2012). Treatment of functional mitral regurgitation by percutaneous annuloplasty: results of the TITAN Trial. Eur J Heart Fail.

[36] Lipiecki J, Siminiak T, Sievert H (2016). Coronary sinus-based percutaneous annuloplasty as treatment for functional mitral regurgitation: the TITAN II trial. Open Heart.

[37] Bail DH (2017). Treatment of functional mitral regurgitation by percutaneous annuloplasty using the Carillon Mitral Contour System-Currently available data state. J Interv Cardiol.

[38] Witte KK, Lipiecki J, Siminiak T (2019). The REDUCE FMR Trial: a randomized sham-controlled study of percutaneous mitral annuloplasty in functional mitral regurgitation. JACC Heart Fail.

[39] Maisano F, Taramasso M, Nickenig G (2016). Cardioband, a transcatheter surgical-like direct mitral valve annuloplasty system: early results of the feasibility trial. Eur Heart J.

[40] Messika-Zeitoun D, Nickenig G, Latib A (2019). Transcatheter mitral valve repair for functional mitral regurgitation using the Cardioband system: 1 year outcomes. Eur Heart J.

[41] Nickenig G, Schueler R, Dager A (2016). Treatment of chronic functional mitral valve regurgitation with a percutaneous annuloplasty system. J Am Coll Cardiol.

[42] Hahn RT, Meduri CU, Davidson CJ (2017). Early feasibility study of a transcatheter tricuspid valve annuloplasty: SCOUT Trial 30-day results. J Am Coll Cardiol.

[43] Suri RM, Vanoverschelde JL, Grigioni F (2013). Association between early surgical intervention vs watchful waiting and outcomes for mitral regurgitation due to flail mitral valve leaflets. JAMA.

[44] Gammie JS, Chikwe J, Badhwar V (2018). Isolated mitral valve surgery: the Society of Thoracic Surgeons Adult Cardiac Surgery Database analysis. Ann Thorac Surg.

[45] Seeburger J, Rinaldi M, Nielsen SL (2014). Off-pump transapical implantation of artificial neo-chordae to correct mitral regurgitation: the TACT Trial (Transapical Artificial Chordae Tendinae) proof of concept. J Am Coll Cardiol.

[46] Colli A, Manzan E, Aidietis A (2018). An early European experience with transapical off-pump mitral valve repair with NeoChord implantation. Eur J Cardiothorac Surg.

[47] Braun D, Nabauer M, Massberg S, Hausleiter J (2019). One-stop shop: simultaneous direct mitral annuloplasty and percutaneous mitral edge-to-edge repair in a patient with severe mitral regurgitation. Catheter Cardiovasc Interv.

[48] Mangieri A, Colombo A, Demir OM (2018). Percutaneous direct annuloplasty with edge-to-edge technique for mitral regurgitation: replicating a complete surgical mitral repair in a one-step procedure. Can J Cardiol.

[49] Grasso C, Attizzani GF, Ohno Y (2014). Catheter-based edge-to-edge mitral valve repair after percutaneous mitral valve annuloplasty failure. JACC Cardiovasc Interv.

[50] Sugiura A, Weber M, Charitos EI, Treede H, Sinning JM, Nickenig G (2020). NeoChord System as an alternative option upon transmitral pressure gradient elevation in the MitraClip procedure. JACC Cardiovasc Interv.

[51] Taramasso M, Feldman T, Maisano F (2018). Transcatheter mitral valve repair: review of the clinical evidence. EuroIntervention.

[52] Rogers JH, Boyd WD, Smith TW, Bolling SF (2018). Early experience with Millipede IRIS transcatheter mitral annuloplasty. Ann Cardiothorac Surg.

[53] Gooley RP, Meredith IT (2015). The Accucinch transcatheter direct mitral valve annuloplasty system. EuroIntervention.

[54] Salmasi MY, Acharya M, Humayun N, Baskaran D, Hubbard S, Vohra H (2016). Is valve repair preferable to valve replacement in ischaemic mitral regurgitation? A systematic review and meta-analysis. Eur J Cardiothorac Surg.

[55] Muller DWM, Farivar RS, Jansz P (2017). Transcatheter mitral valve replacement for patients with symptomatic mitral regurgitation: a global feasibility trial. J Am Coll Cardiol.

[56] Sorajja P, Moat N, Badhwar V (2019). Initial feasibility study of a new transcatheter mitral prosthesis: the first 100 patients. J Am Coll Cardiol.

[57] Sorajja P, Gossl M, Babaliaros V (2019). Novel transcatheter mitral valve prosthesis for patients with severe mitral annular calcification. J Am Coll Cardiol.

[58] Yoon SH, Whisenant BK, Bleiziffer S (2019). Outcomes of transcatheter mitral valve replacement for degenerated bioprostheses, failed annuloplasty rings, and mitral annular calcification. Eur Heart J.

[59] Guerrero M, Vemulapalli S, Xiang Q (2020). Thirty-day outcomes of transcatheter mitral valve replacement for degenerated mitral bioprostheses (valve-in-valve), failed surgical rings (valve-in-ring), and native valve with severe mitral annular calcification (valve-in-mitral annular calcification) in the United States: data from the Society of Thoracic Surgeons/American College of Cardiology/Transcatheter Valve Therapy Registry. Circ Cardiovasc Interv.

[60] Urena M, Vahanian A, Brochet E, Ducrocq G, Iung B, Himbert D (2021). Current indications for transcatheter mitral valve replacement using transcatheter aortic valves: valve-in-valve, valve-in-ring, and valve-in-mitral annulus calcification. Circulation.

[61] Rahhab Z, Ren B, de Jaegere PPT, Van Mieghem N (2018). Kissing balloon technique to secure the neo-left ventricular outflow tract in transcatheter mitral valve implantation. Eur Heart J.

[62] Del Val D, Ferreira-Neto AN, Wintzer-Wehekind J (2019). Early experience with transcatheter mitral valve replacement: a systematic review. J Am Heart Assoc.

[63] Wang DD, Eng MH, Greenbaum AB (2018). Validating a prediction modeling tool for left ventricular outflow tract (LVOT) obstruction after transcatheter mitral valve replacement (TMVR). Catheter Cardiovasc Interv.

[64] Ooms JF WD, Rajani R et al. Computed tomography-derived 3D modeling to guide sizing and planning of transcatheter mitral valve interventions. JACC Cardiovasc Imaging 2021;In Press. 10.1016/j.jcmg.2020.12.034 33744155

[65] Blanke P, Naoum C, Dvir D (2017). Predicting LVOT obstruction in transcatheter mitral valve implantation: concept of the Neo-LVOT. JACC Cardiovasc Imaging.

[66] Meduri CU, Reardon MJ, Lim DS (2019). Novel multiphase assessment for predicting left ventricular outflow tract obstruction before transcatheter mitral valve replacement. JACC Cardiovasc Interv.

[67] de Jaegere P, Rocatello G, Prendergast BD, de Backer O, Van Mieghem NM, Rajani R (2019). Patient-specific computer simulation for transcatheter cardiac interventions: what a clinician needs to know. Heart.

[68] Alharbi Y, Otton J, Muller DWM (2020). Predicting the outcome of transcatheter mitral valve implantation using image-based computational models. J Cardiovasc Comput Tomogr.

[69] Gao H, Qi N, Feng L, et al. Modelling mitral valvular dynamics-current trend and future directions. Int J Numer Method Biomed Eng. 2017;33. 10.1002/cnm.2858 PMC5697636 27935265

